# Disentangling increasing compound extremes at regional scale during Indian summer monsoon

**DOI:** 10.1038/s41598-021-95775-0

**Published:** 2021-08-12

**Authors:** Ravi Kumar Guntu, Ankit Agarwal

**Affiliations:** Department of Hydrology, Indian Institute of Technology Roorkee, Roorkee, 247667 India

**Keywords:** Climate sciences, Hydrology

## Abstract

Compound extremes exhibit greater adverse impacts than their univariate counterparts. Studies have reported changes in frequency and the spatial extent of extremes in India; however, investigation of compound extremes is in the infancy state. This study investigates the historical variation of compound dry and hot extremes (CDHE) and compound wet and cold extremes (CWCE) during the Indian summer monsoon period from 1951 to 2019 using monthly data. Results are analyzed for 10 identified homogeneous regions for India. Our results unravelled that CDHE (CWCE) frequency has increased (decreased) by 1–3 events per decade for the recent period (1977–2019) relative to the base period (1951–1976). Overall, the increasing (decreasing) pattern of CDHE (CWCE) is high across North-central India, Western India, North-eastern India and South-eastern coastlines. Our findings help in identification of the parts of the country affected by frequent and widespread CDHE during the recent period, which is alarming. More detailed assessments are required to disentangle the complex physical process of compound extremes to improve risk management options.

## Introduction

The frequency and spatial extent of weather and climate extremes, such as droughts, floods, heatwaves, and cold waves, are changing due to increased global mean temperatures, land surface feedback, ocean-atmospheric coupling, land-use change, cloud cover and aerosol feedbacks on regional climates^1,2^. These global changes have a significant adverse impact on the economy, eco-hydrological systems, agriculture, and population^3–5^. Several studies^6,7^ over the years have investigated these individual extremes, and significant progress has been made. However, there exists a scope to advance our understanding, especially related to rising multivariate compound extremes. Simultaneous occurrence of two or more natural extremes impacts society greater than their univariate counterparts. For example, the compound dry and hot monsoon during 1951, 1972, 1979, 1987, 2009, 2014, and 2015 in India^8^, compound drought and heatwaves during 2015 in Pakistan and Europe^9^, or the compound coastal flooding in Ravenna, Italy during February 2015^10^ led to more significant impacts on human society, reduction in agricultural productivity and damage to natural ecosystems than those from individual extremes alone.

These multivariate extremes are also referred to as compound extremes/compound events^11^. The IPCC Special Report on Managing the Risks of Extreme Events and Disasters to Advance Climate Change Adaptation defined compound events as ‘(1) two or more extremes occurring simultaneously or successively, (2) combinations of extremes with underlying conditions that amplify the impact of the extremes, or (3) combinations of extremes that are not themselves extremes but lead to an extreme or impact when combined’^3^. Leonard et al. defined the compound event as an extreme-impact event with dependent variables^12^. IPCC in 2012 stated that the investigation on compound extremes received less attention. Indeed, before the IPCC report, a few studies^13,14^ analyzed compound extremes, but it gained significant momentum later across the globe.

Compound extremes associated with precipitation and temperature are most important in hydroclimatology and are commonly studied. The occurrence of compound extremes may be induced due to a common external factor (e.g., changes in regional warming), mutual reinforcement of two extremes (e.g., land surface feedbacks) or conditional dependence of the occurrence of one extreme on to another extreme (e.g., antecedent soil moisture and precipitation conditions for droughts, floods)^15^. On a global scale, Trenberth and Shea showed a strong negative correlation between temperature and precipitation during summer over the continents in both hemispheres^14^. The study concluded that wet summers tend to be associated with cool conditions. In contrast, dry summers tend to be in hot conditions. In most land areas, the correlation between precipitation and temperature is negative during the summer season^16^. Therefore, precipitation deficit (excess) coincides with the high (low) temperature during the summer season.

In the last decade, the dependence between compound precipitation and temperature is investigated at different spatial and temporal scales for diverse climatic regions. These studies used several statistical approaches such as Markov chain model^17^, Quantile regression^18^, Multivariate distribution analyis^19^, Compound indices approach^20^, and Empirical approach^13^. The empirical approach is a framework of simultaneity and has been widely applied in understanding the changes in compound precipitation and temperature extremes at regional and global scales^21,22^. Initially, individual extremes (either using excess over threshold or percentile for a period) are defined, which is then considered in obtaining the count of compound extremes based on the number of co-occurrence of extremes.

Beniston considered 25% and 75% quantiles of precipitation and temperature as the thresholds to define compound extremes across Europe^13^. Further, Hao et al., offered global analysis and revealed an increase in the frequency of joint warm/dry and warm/wet conditions during 1951–2004^21^. Considering a recent dataset, Wu et al. reported a more frequent and widespread spatial extent of the compound dry and hot conditions during the summer and winter seasons across China^22^. Zscheischler and Fischer recently investigated the record-breaking compound dry and hot conditions of 2018 during the summer season in Germany and projected that compound extreme would become more likely^23^. In summary, these aforementioned literature highlights a significant increase in the compound dry and hot summers at multiple spatiotemporal scales worldwide, including the Indian subcontinent^24,25^.

From the beginning of the twenty-first century, different parts of the Indian subcontinent suffered from regional drought and excess precipitation, causing catastrophic impacts on society and ecosystems. Economically, $80 billion losses have incurred due to climate-related hazards from 1998 to 2017, along with irretrievable damages to thousands of human lives^26^. Mishra et al. reported crop yield has significantly reduced 146 and 111 kg/ha due to compound dry and hot summers in 1987 and 2009, respectively^8^. Sharma and Mujumdar investigated changes in the frequency of compound droughts and heatwaves over homogenous regions of India defined by the Indian Meteorological Department (IMD)^27^. The findings are indeed useful and contributed significantly to our existing knowledge. However, owing to the spatial diversity of precipitation over India and its emerging changes, we argue that homogenous regions developed by IMD do not account for the spatiotemporal variability of precipitation^28^. Previous studies^29,30^ pointed out that IMD homogeneous regions lack coherent climate conditions. Very recently, Guntu et al. developed homogenous regions by accounting for temporal variability and precipitation magnitude at multiple time scales^31^. This means that homogeneous regions are defined based on climate variability at different time scales (monthly, seasonal and annual) rather than mean climate conditions. As a result, these homogenous regions are robust and coherent in common seasonality, climate sensitivity, and global mechanisms that drive variability for different regions. However, studies of systematic investigation of frequency and the spatial extent of different types of compound extremes over homogenous regions of India are relatively rare. A comprehensive analysis of compound extremes will help identify and disentangle the regions prone to increased likelihood during the Indian summer monsoon (ISM). Therefore, this paper aims to analyze the temporal and spatial characteristics of compound extremes of monthly temperature and precipitation to evaluate the historical variation of frequency and spatial extent of compound dry and hot extremes (CDHE) and compound wet and cold extremes (CWCE) during the Indian summer monsoon (ISM) across homogeneous regions of India.

## Study area and data

**Figure 1:**
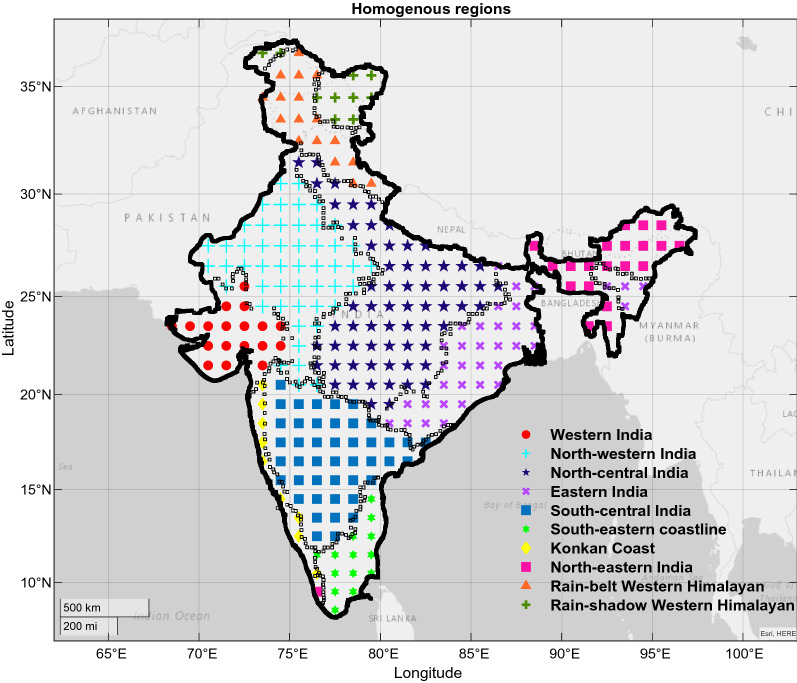
Geographical representation of grid points enclosed in each homogenous region. As per Koppen climate classification and accounting for the temporal variability of precipitation, Guntu et al. classified Western and North-western India as semi-arid and desert, North-central India as humid subtropical, Eastern India as tropical savannah, South-central India as semi-arid, South-eastern coastline as warm and humid subtropical, Konkan coast as tropical monsoon, North-eastern India as humid subtropical, North India (from Western Himalayas to Eastern Himalayas) has Alpine climate regime^31^﻿. This figure is generated using Mapping Toolbox in MATLAB. (Ver 9.9 https://in.mathworks.com/products/mapping.html).

The Indian subcontinent lies approximately between 8°–37° N latitude and 68°–98° E longitude, covering 3,287,590 km^2^. We use a long-term gridded dataset (1951–2019) of daily precipitation and temperature developed by the IMD for the spatial domain of 66° E to 98° E and 8° N to 37° N, covering the mainland region of India. The spatial resolution of the data is 1° × 1° in latitude and longitude direction, and the geographical representation of grid points is shown in Fig. 1 (see Table S1 for the number of grid points falling in each homogenous region). Precipitation and temperature dataset are generated from a diverse network of 1803 and 395 gauging stations across India using an Inverse distance weighted (IDW) interpolation scheme^32,33^. Both the datasets have been extensively used in earlier studies^34,35^ for various hydro-meteorological applications and confirm that the data is highly accurate and capable of capturing the country's spatiotemporal diversity of precipitation and temperature and can be retrieved from Ref.^36^.

India’s climate shows a significant variation across a vast geographic scale and varied topography, making regionalization tricky. In the past several studies^29,30^ had contributed to the development of homogenous regions. Guntu et al., proposed a regionalization framework and discussed that precipitation magnitude and temporal variability at multiple timescales should be accounted for improved identification of homogenous regions^31^. The identified ten homogenous regions are not subjective rather an objective approach that considers intraregional homogeneity and interregional heterogeneity of grid points during the formation of regions. In addition, an optimal number of clusters is identified based on the Silhouette width, which can specify the degree of similarity of grid points inside a cluster (cohesion) compared to other clusters (separation). The average Silhouette width revealed that a probable number of clusters are 6, 10, or 14. It is detected that on working with 14 clusters, some of the clusters have very few (one or two) stations. On the other side, considering six clusters, it is noticed that the interregional heterogeneity was missing, i.e. properties of grid points within one cluster were matching the grid points in other clusters. On selecting 10 clusters (see Fig. 1), the intraregional homogeneity and interregional heterogeneity of grid points were maintained (for more details, see Sects. 3.6 and 4.2 in Ref.^31^).

## Methodology

### Definition of compound extremes

**Figure 2:**
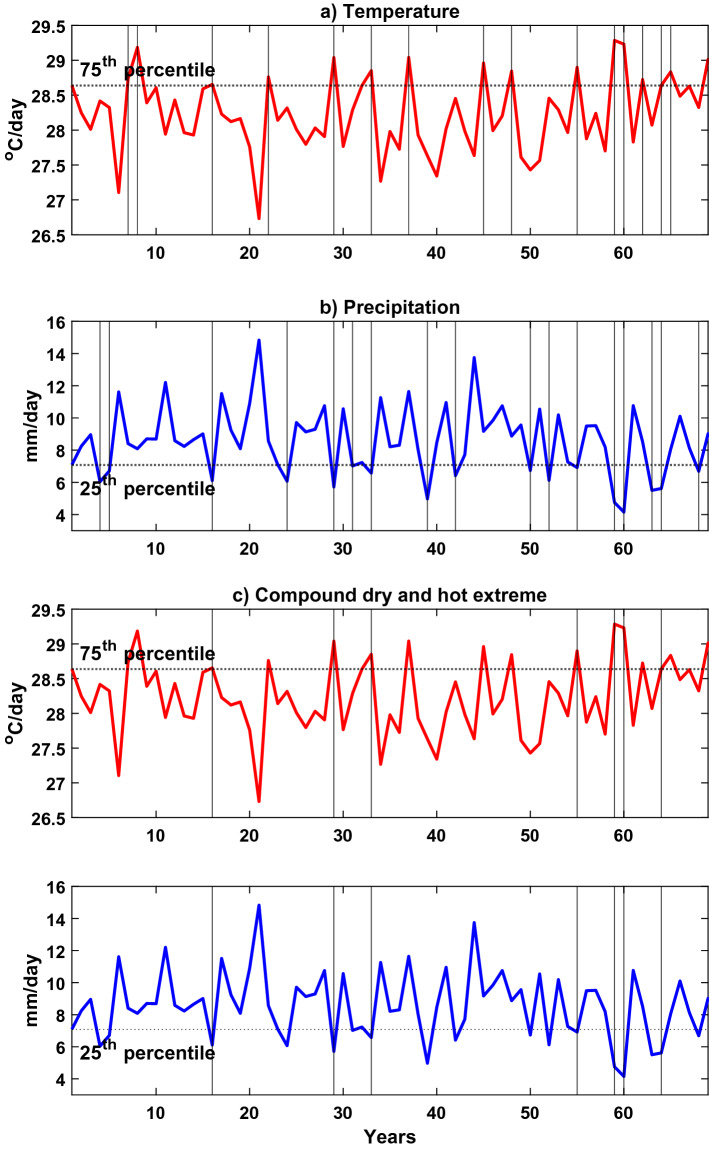
Illustration of a typical compound dry and hot extreme (**c**) and its comparison with univariate events (**a**) Temperature > 75th percentile and (**b**) Precipitation < 25th percentile. The vertical and horizontal lines are shown to visualize an event and the threshold used to define the event).

The 25% and 75% quantiles of monthly precipitation and temperature are used as threshold levels for defining the compound extremes at each grid point (see Fig. 2a and b). Following^21^, the combination P < 25/T > 75 and P > 75/T < 25 represents two climate combinations: warm/dry and cold/wet, respectively. In other words, compound extremes are defined as being simultaneously in an outer quartile of both precipitation and temperature (see Fig. 2c). Following Hao et al., ‘extreme’ in the present study is defined as a relatively modest departure from the mean value, i.e., above 75 quantiles or below 25 quantiles^21^. The reason for selecting 25 and 75 as the thresholds is that they provide sufficiently large sample sizes for robust statistical assessments^37^. While events in the lower tail of the distribution are expected to cause a reduction in agricultural productivity, these moderate extremes also impact relevance^37^. Furthermore, since typical compound extremes persist for several days, the monthly scale is more appropriate for medium-term (from few years to few decades) water resources planning and management compared to a 3-day to pentad timescale^38^. Therefore, in the present study, the monthly timescale could be considered a compromising timescale between the relatively coarser and finer temporal resolutions.

### Frequency of compound extremes

Changes in the compound extremes frequency during 1977–2019 (referred to as the recent period) are compared with 1951–1976 (referred to as base period) at each grid point. We have selected 1977 to separate two time periods based on the studies on the climate shift around 1976–1977 and its implication to Indian summer monsoon^39,40^. As the length of the two selected periods is not equal, the frequency changes are expressed per decade. The frequency per decade during a time window is calculated as follows [see Eq. (1)]

1 $${frequency} \, {per} \, { decade}_{{time} \, { window}}=\frac{{Number } \, {of } \, {months } \, {shown } \, {with } \, {occurence } \, {of} \, { compound } \, {extreme} \, { during} \, { a} \, { particular } \, {time} \, { window}}{{Number} \, { of} \, { decades}}$$

In Eq. (1), the number of decades during the base period and the recent period is 2.6 and 4.3, respectively. Next, the changes in frequency per decade is calculated as follows [see Eq. (2)]

2 $$Change \, in \, frequency \,per \, decade = \frac{{frequency \, per \, decade_{{recent \, period}} - frequency \, per \, decade_{{base \, period}} }}{{frequency \, per \, decade_{{base \, period}} }}$$

The change in frequency per decade of compound extremes based on IMD observations at every grid point between two periods is tested for statistical significance using a two-tailed t-test hypothesis testing against the null hypothesis of no difference.

### Spatial extent of compound extremes

Compound extreme’s impact is not limited to a grid point; instead, the same event extended to a large region. Spatial extent is the ratio of the number of grid points affected due to compound extreme to the total number of grid points in a homogenous region. For example, during June 1951, if ten grid points have a CDHE occurrence out of 20 grid points, then the spatial extent of compound extreme for 1951 is 0.5 (10/20). In continuation, if two grid points have a CWCE scenario in the same year, then the spatial extent of CWCE in 1951 is 0.1 (2/20). Non-parametric MK test^41,42^ is employed to detect the statistical significance in the historical variation of spatial extent trends against the null hypothesis of no trend (for mathematical equations, see Text S1 in the Supplementary Material).

A previous study by Sharma and Mujumdar, defined the spatial extent as the number of grid points to India’s total number of grids^27^. However, averaging over the entire Indian landmass underestimates the spatial extent changes at the regional scale. Therefore, in this study, the spatial extent time series is constructed for every homogenous region to detect the trend and spatial extent changes during ISM.

## Results and discussion

Section “Changing pattern of precipitation and temperature” reports trend analysis of monthly precipitation and monthly temperature during ISM over the period 1951–2019 based on the MK test. Section “Changes in the frequency of compound extremes” links changes in the frequency of compound dry and hot extremes (CDHE) and compound cold and wet extremes (CWCE) for the recent period (1977–2019) relative to the base period (1951–1976) with changing pattern of precipitation and temperature. Section “Changes in the spatial extent of compound extremes” discusses the significant changes in the probability distribution of CDHE and CWCE spatial extent towards identifying homogenous regions prone to increased likelihood.

### Changing pattern of precipitation and temperature

**Figure 3:**
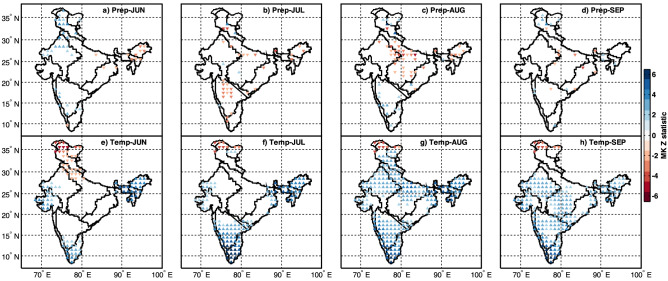
Geographical representation of trends in the monthly precipitation (**a–d**) and temperature (**e**–**h**) in Indian summer monsoon (June, July, August and September) of 1951–2019 across the homogenous regions of India. Statistical significance is conducted using the non-parametric Mann–Kendall (MK) test. Grid points with a 95% confidence level are only indicated with triangles. The Upper and Lower triangles indicate increasing and decreasing trend, respectively. Colorbar represents variation in the MK Z statistic. This figure is generated using Mapping Toolbox in MATLAB R 2020b (Version 9.9 https://in.mathworks.com/products/mapping.html).

Long-term trends for every grid point across India are analyzed for monthly precipitation (Fig. 3a–d) and mean temperature (Fig. 3e–h), respectively. Grid points showing a trend with a 95% confidence level based on the MK test are only indicated in Fig. 3. Monthly precipitation during June (Fig. 3a) exhibits a positive trend for North-western India, Konkan coast and a declining trend for North-eastern India. This observation is consistent with Subash and Sikka^34^. They also reported a similar changing precipitation pattern across Western India using the Indian Institute of Tropical Meteorological (IITM) monthly precipitation dataset. July precipitation (Fig. 3b) exhibits a decreasing trend for South-central India and the Himalayas' foothills. A similar observation is reported by Taxak et al., using a CRU (0.5^0^ × 0.5^0^) gridded precipitation dataset^43^. Most notably, August precipitation (Fig. 3c) is reducing significantly for North-central India and North-eastern India. Simultaneously, it is increasing along the lee-side of the Western Ghats and some parts of South-central India.

Several studies^44,45^ has reported that ISM is declining in the monsoon's core region. A few scientific evidence reported are (1) largescale decrease in the transport of moisture from the Arabian sea along the west coast onto the mainland, called weakening of monsoon circulation due to the eastern equatorial Indian Ocean's rapid warming^45^, (2) Increase of aerosol emissions and anthropogenic greenhouse gases led to the decrease in surface solar radiation over south Asia, by this means mainland surface becomes cool leading to a reduction in thermal contrast between the North and south Indian ocean, causing weakening of tropical circulation (zonal component)^46^, (3) High-resolution atmospheric model confirms a poleward shift in ISM synoptic activity due to a warmer climate, resulting in a reduction in the travel of low-pressure systems across North-central India^47^, (4) Naidu et al., showed a decrease in the strength of Tropical Eastery jet streams due to global warming as the evidence, and claimed monsoon activity is becoming insufficient due to the reduction in the number of low pressure systems^48^.

Interestingly, it was revealed that decreasing precipitation is mainly confined to August during the ISM, while the contribution of June, July and September remains constant for North-central India. The pattern found in this month illustrates a decrease of active monsoon conditions over central India in August. During September (Fig. 3d), monthly precipitation is not showing any significant changing pattern across India. The precipitation trends (JJAS) agree with the previous studies concerning monthly precipitation trends across India^34,35,49–53^.

Temperature trend analysis reveals an increase in the mean temperature across Western India, South-eastern coastline, North-eastern India during June (Fig. 3e), and a decreasing trend for Rain-belt Western Himalayan. Figure 3f shows July temperature is increasing for Western India, South-central India, South-eastern coastline, Eastern India and North-eastern India. The temperature is rising for every homogenous region to a large spatial extent during August (Fig. 3g), except in Eastern India. Most notably, for north-central India, a rising temperature is coinciding with decreased precipitation is not reported earlier. It gives an emerging picture of potential water shortage if it continues, coinciding with major crop growth season threatens the nation's food security region. September temperature (Fig. 3h) shows an increasing trend across India except for western Himalayan and north-western India, North-central India, Eastern India. While the Karakorum range has shown a decreasing trend in monthly temperature during July (Fig. 3f), August (Fig. 3g) and September (Fig. 3h) despite the warming climate. The findings of changing precipitation and temperature patterns for Rain-belt Western Himalayan agree with the previous studies^54,55^.

For the country as a whole, the number of grid points shown with changing temperature pattern are greater than precipitation due to rising warm climatic conditions. Praveen and Sharma recently evaluated the historical variation of crop productivity using multiple regression analysis based on precipitation and temperature^56^. They concluded that crop production (tea, jowar, maize, ragi, sugarcane, and wheat) has reduced over the period 1967–2016, majorly due to increased temperature. This means that changing temperature pattern plays a key role along with other factors of the hydrological cycle.

The changing monthly temperature pattern is consistent with findings of the previous studies^34,35,57,58^. Unlike those studies, the present study used long-term historical data and explored trend analysis over homogeneous regions based on coherent climatic conditions. Assessment of the changes based on the recent and long-term good quality data is more reasonable for decision-making. It offers a reliable state of precipitation and temperature concerning climate variability and change.

### Changes in the frequency of compound extremes

**Figure 4:**
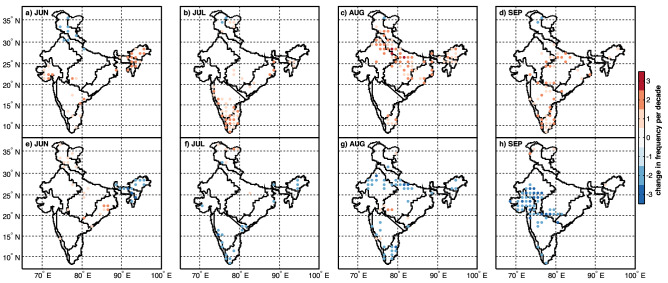
Spatial distribution of change in frequency per decade in the occurrence of the CDHE (Top panel, **a**–**d**) and CWCE (Bottom panel, **e**–**h**) for the recent period (1977–2019) relative to the base period (1951–1976) during Indian summer monsoon months across homogenous regions of India. Colorbar represents variation in the change in frequency per decade. This figure is generated using Mapping Toolbox in MATLAB R 2020b (Version 9.9 https://in.mathworks.com/products/mapping.html).

As mentioned before, the complete time duration of the study, i.e. 1951–2019, was divided into the recent period (1977–2019) and base period (1951–1976) to investigate the change in frequency per decade. The change in frequency per decade of compound extremes based on IMD observations at every grid point between two periods is tested for statistical significance using a two-tailed t-test hypothesis testing against the null hypothesis of no difference. Figure 4 shows the spatial distribution of grid points across homogeneous regions rejected at a 95% confidence level. The number of CDHE and CWCE occurrences for two periods (recent, base) are also provided in Figs. S1 and S2.

#### Compound dry and hot extreme (CDHE)

CDHE (low precipitation and high temperature) is of specific concern because of their mutual dependence in compound drought, causing significant damage to eco-hydrological systems^8^. Figure 4a–d displays the recent (1977–2019) change in frequency per decade of CDHE relative to the base period (1951–1976).

Our analysis based on IMD revealed that the occurrence of compound dry and hot extremes had increased significantly in recent decades compared to the base period. Interestingly, the increment is not uniform over different months and regions. For instance, during June (Fig. 4a), the occurrence of CDHE has increased by 1 to 2 events per decade during the recent period compared to the base period for western India, North-eastern India (Rain-belt Western Himalayan). For the July month, the frequency has increased by 1–3 events per decade over South-eastern coastline, Konkan coast and parts of South-central India, Eastern India, Rain-shadow region and North-central India (Fig. 4b). The frequency has increased 1 to 3 events per decade compared to the base period for North-central India, North-eastern India, some parts of North-western India, Rain-belt Western Himalayan, Eastern India, and South-eastern coastline during August (Fig. 4c). Therefore, the increasing temperature and distribution of precipitation during the monsoon season should be accounted for the widespread trend. In support of that, Kothawale and Rupa Kumar had shown a strong negative simultaneous correlation between precipitation and temperature during the monsoon season^59^.

On the other hand, Kothawale et al. concluded that Indian mean temperatures are strongly correlated with sea surface temperature (SST) in the eastern pacific and the equatorial Indian Ocean^60^. Guntu et al. developed the Standardized Variability Index (SVI) to quantify the randomness of the temporal distribution of precipitation based on annual time series and the total sum of precipitation^61^. Using entropy-based SVI, they highlighted that the distribution of precipitation during monsoon is getting narrower during the past decades. They implied that the precipitation amount is getting concentrated to starting months of the season, leaving the latter part of the season in drier condition. Therefore, the temperature is significantly rising during August and September due to the strong negative correlation and SST warming. As a result, it increases the likelihood of compound extremes across North-central India.

#### Compound wet and cold extreme (CWCE)

Our analysis concerning CWCE (high precipitation and low temperature) highlight that the frequency has decreased, in general, for most of the summer monsoon months except June. June (Fig. 4e) shows the changes in frequency per decade by 1–2 events across Rain-belt Western Himalaya and parts of Eastern India, North-central India. During July (Fig. 4f), the frequency has decreased by 1 to 2 events per decade across the Konkan coast, North-eastern India, South-eastern coastline and South-central India. Further, a widespread decrease was revealed in North-central India (Fig. 4g) and Western India (Fig. 4h) during August and September.

#### Linkages of CDHE and CWCE to changing patterns of precipitation and temperature

We have further discussed the changing pattern in the frequency of compound extremes for every homogenous region by linking it to precipitation and temperature patterns (Fig. 3). For western India, the increasing frequency of CDHE during June (Fig. 4a) is rising due to temperature (Fig. 3e). Also, the decrease in the frequency of CWCE (Fig. 4h) is attributable to a rise in temperature (Fig. 3h). For North-western India, an increase in the frequency of CDHE during August (Fig. 4c) is attributable to decreasing precipitation (Fig. 3c) as well as an increasing temperature (Fig. 3g). In comparison, the decreasing frequency of CWCE (Fig. 4g) is primarily attributable to the rising temperature (Fig. 3g). For North-central India, an increase in the frequency of CDHE during August and September (Fig. 4c,d) is attributable to both decreasing precipitation (Fig. 3c) and increasing temperature (Fig. 3g).

As for CWCE, a decrease in the frequency during August (Fig. 4g) is attributable to both the changing pattern of precipitation (decreasing, Fig. 3c) as well as temperature (increasing, Fig. 3g). On the contrary, increasing temperature alone (Fig. 3h) has decreased the frequency during September (Fig. 4h). For South-central India, the increase in the frequency of CDHE during July (Fig. 4b) is due to changing temperature only. For South-eastern coastlines, a significant increase in the frequency of CDHE during July (Fig. 4b), August (Fig. 4c) and September (Fig. 4d) are driven by an increase in temperature alone (Fig. 3f,g,h). For North-eastern India, an increase in the frequency of CDHE during June (Fig. 4a) and August (Fig. 4c) is attributable to both decreases in precipitation and an increase in temperature. Similarly, a decrease in the frequency of CWCE during June (Fig. 4e) and August (Fig. 4g) is attributable to both the changing pattern of precipitation and temperature. For the Rain-belt Western Himalayan (see Fig. 1), CDHE (CWCE) frequency has decreased (increased) during June (Fig. 4a,e), which is deviating from the rest of the homogenous regions. The changes in compound extremes can be attributed to changing precipitation patterns (increase in Fig. 3a) and temperature (decrease in Fig. 3e). Sharma and Mujumdar reported an increasing trend in droughts' co-occurrence with longer heatwaves for western India, Central India and Peninsular India^27^. Further, Mishra et al., reported seven CDHE between 1951 and 2018, considering All-India monsoon precipitation and temperature^8^. Out of these seven CDHE, three have occurred in the past decade, indicating an increase in frequency to 3 events per decade. Our results on the change in frequency per decade agree with the previous study. Unlike the prior study, detailed regional information on the frequency and spatial extent changes are discussed in the following section.

### Changes in the spatial extent of compound extremes

Spatial extent affected by compound extremes within a homogenous region is extracted for each year to characterize the temporal evolution of compound extreme. The spatial extent is the areal fraction of homogenous region shown with either CDHE or CWCE in a particular month during a year (see “Spatial extent of compound extremes”). The temporal evolution of the spatial extent of CDHE and CWCE during a specific month (JJAS) for every homogenous region is provided in Figs. S3 and S4. MK test is employed to detect the statistical significance in spatial extent trends against the null hypothesis of no trend. Significance test results for CDHE and CWCE during JJAS for every homogenous region are given in Tables 1 and 2, respectively.

**Table 1:** MK test result for each homogenous region's CDHE spatial extent during the Indian summer monsoon months.

Cluster	JUN		JUL		AUG		SEP	
	${Z}_{s}$	${\mathrm{p}}_{\mathrm{v}}$	${Z}_{s}$	${\mathrm{p}}_{\mathrm{v}}$	${Z}_{s}$	${\mathrm{p}}_{\mathrm{v}}$	${Z}_{s}$	${\mathrm{p}}_{\mathrm{v}}$
Western India	**2.04**	**0.04**	0.55	0.58	0.69	0.49	0.74	0.46
North-western India	0.83	0.41	0.70	0.48	**3.11**	**0.00**	1.66	0.10
North-central India	− 0.3	0.76	0.79	0.43	**3.67**	**0.00**	**2.42**	**0.02**
Eastern India	1.51	0.13	**3.38**	**0.00**	**2.37**	**0.02**	**2.71**	**0.01**
South-central India	1.25	0.21	**2.62**	**0.01**	**2.10**	**0.04**	**2.01**	**0.04**
South-eastern coastline	1.63	0.10	**3.62**	**0.00**	**2.21**	**0.03**	**2.65**	**0.01**
Konkan Coast	1.03	0.30	**2.04**	**0.04**	1.55	0.12	1.41	0.16
North-eastern India	**2.15**	**0.03**	**2.91**	**0.00**	**2.86**	**0.00**	**2.23**	**0.03**
Rain-belt western Himalayan	− 1.54	0.12	− 1.26	0.21	− 0.50	0.62	− 0.92	0.36
Rain-shadow western Himalayan	− 1.33	0.18	0.56	0.57	− 0.18	0.86	− 0.68	0.49

${\mathrm{Z}}_{\mathrm{s}}$ is the Mann–Kendal Z statistic; ${\mathrm{p}}_{\mathrm{v}}$ is the p-value corresponding to ${\mathrm{Z}}_{\mathrm{s}}$; Bold text has rejected the null hypothesis, i.e. there is a significant trend in spatial extent at a 95% confidence level (p-value ≤ 0.05, see Text S1).

**Table 2:** Same as Table 1 but for CWCE spatial extent.

Cluster	JUN		JUL		AUG		SEP	
	${Z}_{s}$	${\mathrm{p}}_{\mathrm{v}}$	${Z}_{s}$	${\mathrm{p}}_{\mathrm{v}}$	${Z}_{s}$	${\mathrm{p}}_{\mathrm{v}}$	${Z}_{s}$	${\mathrm{p}}_{\mathrm{v}}$
Western India	− 0.70	0.48	− 0.79	0.43	− 1.61	0.11	− **2.58**	**0.01**
North-western India	1.07	0.29	− 0.24	0.81	− **2.63**	**0.01**	− **2.44**	**0.01**
North-central India	1.26	0.21	− 0.47	0.64	− **3.09**	**0.00**	− **2.55**	**0.01**
Eastern India	0.23	0.82	− 1.88	0.06	− **2.14**	**0.03**	− **2.07**	**0.04**
South-central India	0.14	0.89	− **1.99**	**0.05**	− **2.40**	**0.02**	− **2.07**	**0.04**
South-eastern coastline	− 0.53	0.60	− **2.89**	**0.00**	− **2.51**	**0.01**	− 1.80	0.07
Konkan Coast	− 0.62	0.53	− **2.17**	**0.03**	− **2.66**	**0.01**	− **2.78**	**0.01**
North-eastern India	− **3.77**	**0.00**	− **2.78**	**0.01**	− **2.71**	**0.01**	− 1.45	0.15
Rain-belt western Himalayan	**2.25**	**0.02**	0.05	0.96	− 1.01	0.31	− 0.40	0.69
Rain-shadow western Himalayan	1.06	0.29	0.43	0.67	0.51	0.61	0.10	0.92

#### Spatial extent of CDHE

From Table 1, an increasing trend in the spatial extent of CDHE is observed for Western India and North-eastern India during June. During July, homogenous regions with the increasing trend are Eastern India, South-Central India, South-eastern coastline, Konkan coast and North-eastern India. Furthermore, a higher number of homogenous regions have rejected the null hypothesis and are shown to increase in spatial extent during August and September.

#### Spatial extent of CWCE

From Table 2, the spatial extent of CWCE is significantly decreasing for North-eastern India. In comparison, an increasing trend is shown for Rain-belt Western Himalayan. During July, homogenous regions with a significant decreasing trend in spatial extent are South-Central India, South-eastern coastline, Konkan coast and North-eastern India. Further, the South-eastern coastline has the lowest Z-statistic value, demonstrating a decrease in the spatial extent during recent decades. During August and September, homogenous regions with a significant decreasing trend in spatial extent are seven and six out of ten homogenous regions of India, revealing a significant increase in warm conditions.

#### Spatial extent of CDHE and CWCE for one particular homogeneous region

**Figure 5:**
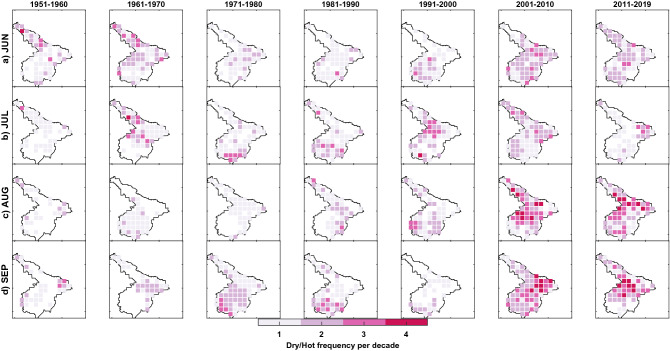
Geographical representation of spatial extent over homogenous North-Central region during (**a**) June, (**b**) July, (**c**) August and (**d**) September at decadal time scale. Colorbar represents the number of months shown with the occurrence of CDHE during the decade at each grid point. This figure is generated using Mapping Toolbox in MATLAB R 2020b (Version 9.9 https://in.mathworks.com/products/mapping.html).

To further understand and visualize the changing pattern of spatial extent for a particular homogeneous region, the number of occurrences of CDHE and CWCE at every grid point is plotted for the last seven decades. Figure 5 demonstrates the increase in the spatial pattern of CDHE for North-central India over the last seven decades. For brevity purposes, remaining homogenous regions are provided in the Figs. S5–S11. For North-central India (see Fig. 5), the spatial extent of CDHE has significantly increased during the last decades. There is a consistent increase in the spatial extent for August (Fig. 5c) and September (Fig. 5d) when compared to June (Fig. 5a) and July (Fig. 5b). The frequency has also increased by two to three times for most grid points, which is in line with the evidence shown in Fig. 4. A similar pattern (increase in frequency and widespread spatial extent of CDHE) has been exhibited for most homogenous regions in the last two decades, which is alarming.

**Figure 6:**
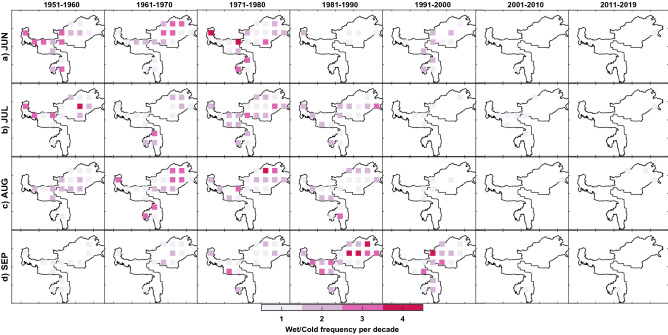
Geographical representation of spatial extent over North-eastern homogenous region during (**a**) June, (**b**) July, (**c**) August and (**d**) September. Colorbar represents the number of months shown with the occurrence of CWCE during the decade at each grid point. This figure is generated using Mapping Toolbox in MATLAB R 2020b (Version 9.9 https://in.mathworks.com/products/mapping.html).

Figure 6 demonstrates the decrease in the spatial pattern of CWCE for North-eastern India over the last seven decades (see Figs. S12–S18 for remaining regions). For North-eastern India (see Fig. 6), the spatial extent of CWCE has significantly decreased during the last two decades. The frequency has also reduced by two to three times S18for the last decade, revealing zero wet and cold conditions during the Indian summer monsoon for north-eastern India. Based on a visual inspection, if a similar pattern continues with the same rate, it will lead to zero CWCE for most homogenous regions by 2030 (Figs. S12–). These findings suggest that the joint extremes' spatial extent related to warm mode has increased while cold mode has decreased in India during the Indian summer monsoon.

#### Cumulative distribution function of spatial extent

**Figure 7:**
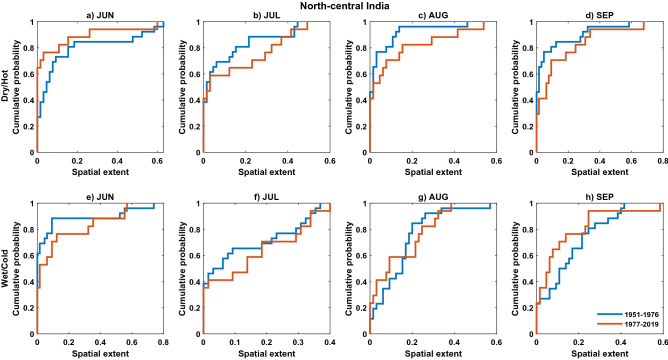
Empirical CDF of CDHE (**a**–**d**) and CWCE (**e**, **f**) spatial extent during June, July, August and September for the recent period (1977–2019) versus base period (1951–1976) for North-central India. This figure is generated in MATLAB R 2020b (Version 9.9 https://in.mathworks.com).

To better visualize the statistical characteristics of compound extremes, we explored the cumulative distribution function of spatial extent for the recent period (1977–2019) versus the base period (1951–1976). Following Sharma and Mujumdar, eCDF (empirical cumulative distribution function) of the spatial extent of CDHE (dry/hot) and CWCE (wet/cold) is plotted (Fig. 7) for the recent period and base period^27^. A two-sample Kolmogorov–Smirnov (KS) test, a non-parametric test, is employed to test the significant difference in the eCDF between the two periods (for mathematical equations, see text S2 in the supplementary material). In the present study, outliers are not removed because of their strong influence in sociological terms. Sharma and Mujumdar suggested preserving the outliers to investigate the nature of recent extremes to those of base period^27^. The present study hypothesizes that recent and base periods' extent values come from the same underlying continuous population. Results from the hypothesis testing are given in Table 3 to summarize the results, where H = 0 (1) states the hypothesis is accepted (rejected) at a 95% confidence level.

**Table 3:** A two-sample KS test result for the spatial extent of CDHE before and after 1976 for each homogenous region.

Cluster	JUN		JUL		AUG		SEP	
	${Z}_{s}$	${\mathrm{p}}_{\mathrm{v}}$	${Z}_{s}$	${\mathrm{p}}_{\mathrm{v}}$	${Z}_{s}$	${\mathrm{p}}_{\mathrm{v}}$	${Z}_{s}$	${\mathrm{p}}_{\mathrm{v}}$
Western India	0	0.20	0	1.00	0	1.00	0	0.73
North-western India	0	0.91	0	1.00	**1**	**0.00**	**1**	**0.04**
North-central India	0	0.26	0	0.97	**1**	**0.00**	**1**	**0.03**
Eastern India	0	0.72	**1**	**0.01**	0	0.15	0	0.15
South-central India	0	0.30	0	0.07	0	0.09	0	0.06
South-eastern coastline	0	0.29	**1**	**0.01**	0	0.23	1	0.02
Konkan Coast	0	1.00	0	0.07	0	0.43	0	0.38
North-eastern India	0	0.08	0	0.17	0	0.08	0	0.29
Rain-belt western Himalayan	0	0.06	**1**	**0.02**	0	1.00	0	0.17
Rain-shadow western Himalayan	0	0.19	0	1.00	0	1.00	0	0.40

H is the hypothesis (i.e. two samples are from the same underlying continuous population; ${\mathrm{p}}_{\mathrm{v}}$ is the p-value corresponding to H; H = 0 (1) state hypothesis is not rejected (rejected) at the 5% significance level; Bold text represent significant values greater than 95% confidence level (p-value $\le$ 0.05, see Text S2).

For CDHE (top panel, Fig. 7) eCDF is plotted to illustrate the distribution of spatial extent values for the recent period (red line) and base period (blue line) for one particular homogenous region (North-central India). The additional supporting analysis for the rest of the homogenous region is shown in the supplementary information for brevity purposes (Figs. S19–S27). For North-central India, the hypothesis is rejected for August and September (see Table 3), which states that spatial extent values of the recent period are significantly different from the base period values. The mean of the spatial extent has significantly increased in the recent period relative to the base period during August (Fig. 7c) and September (Fig. 7d). Also, the upper tails of the eCDF during 1977–2019 were longer than 1951–1976, signifying compound dry and hot events affecting large spatial areas have increased during 1977–2019. Similar changes in the statistical characteristics of spatial extent are exhibited for North-western India (Fig. S20). There is no significant difference in the statistical characteristics of spatial extent values for the rest of the homogenous regions during August and September (Table 3). Similarly, for June, none of the homogenous regions has a significant difference. However, the statistical characteristics of the spatial extent for Eastern India, South-eastern coastline, and Rain-belt Western Himalayan are significantly different in recent periods at a 95% confidence level during July (see Table 3).

For CWCE (bottom panel in Figs. 7, S19–S27), during June, North-eastern India has shown a significant change in the statistical characteristics of spatial extent in the recent period (see Table 4). In July, the South-eastern coastline has rejected the hypothesis at a 95% confidence level, and rest of the homogenous regions accepted the hypothesis (see Table 4). In August, the hypothesis is rejected for North-western India, North-central India, South-eastern coastline, Konkan coast and North-eastern India. North-central India (Fig. 7g) has shown a significant change in the mean of spatial extent in the recent period and rejected the hypothesis (see Table 4). Also, higher spatial extent values during the base period have reduced in the recent period. For September (see Table 4), there is a significant difference in the statistical characteristics (rejection of hypothesis) at a 95% confidence level for Western India, North-western India, North-central India, South-central India and the Konkan coast. The results indicate that more areas are covered by compound extremes related to warm mode while fewer areas are covered by the Indian summer monsoon's cold mode during recent decades.

**Table 4:** Same as Table 3 but for CWCE.

Cluster	JUN		JUL		AUG		SEP	
	${Z}_{s}$	${\mathrm{p}}_{\mathrm{v}}$	${Z}_{s}$	${\mathrm{p}}_{\mathrm{v}}$	${Z}_{s}$	${\mathrm{p}}_{\mathrm{v}}$	${Z}_{s}$	${\mathrm{p}}_{\mathrm{v}}$
Western India	0	0.86	0	1.00	0	0.55	**1**	**0.01**
North-western India	0	0.99	0	0.94	**1**	**0.03**	**1**	**0.05**
North-central India	0	0.30	0	1.00	**1**	**0.03**	**1**	**0.02**
Eastern India	0	0.73	0	0.56	0	0.09	0	0.28
South-central India	0	0.92	0	0.22	0	0.33	**1**	**0.05**
South-eastern coastline	0	1.00	**1**	**0.01**	**1**	**0.03**	0	0.71
Konkan Coast	0	1.00	0	0.08	**1**	**0.03**	**1**	**0.00**
North-eastern India	**1**	**0.00**	0	0.06	**1**	**0.01**	0	1.00
Rain-belt western Himalayan	0	0.26	0	0.88	0	0.61	0	1.00
Rain-shadow western Himalayan	0	0.79	0	0.88	0	0.68	0	1.00

## Conclusions

We investigated the regional changes in the frequency and spatial extent of compound dry and hot extremes (CDHE) and compound wet and cold extremes (CWCE) during Indian summer monsoon (ISM). The ten homogenous regions derived from multi-scale standardized variability index and self-organizing map are considered from our previous study^31^ to characterize the CDHE (CWCE) patterns at a regional scale. Our findings revealed that the frequency of CDHE (CWCE) has increased by 1–3 events per decade (decreased by 1–2 events per decade) for the recent period (1977–2019) relative to the base period (1951–1976). This increasing (decreasing) pattern of CDHE (CWCE) is high across North-central India, Western India, North-eastern India and South-eastern coastlines. The increasing frequency of CDHE is mainly attributed to the Indian Ocean SST variability post-1976/1977 climate shift. Several studies concluded that the influence of equatorial Pacific Ocean SST has decreased, and eastern equatorial Indian Ocean SST has increased after the climate shift leading to an early withdrawal of ISM. As a result, the length of ISM has shrunk, causing an increased (decreased) frequency of CDHE (CWCE). Therefore, considering the joint conditions of hydro-climatic variables and multivariate modelling is essential to advance informed understanding of compound extremesclimate change adaptation. Moreover, identified regions with an increased likelihood of compound dry and hot extremes are critical for risk management strategies. Though the present study has contributed significantly to understanding CDHE (CWCE), the dynamics of compound extremes could be further investigated at various temporal resolutions and at different time scales for present and future datasets.

## Supplementary Information

Supplementary Information.

## References

[1] AghaKouchak A (2020). Climate extremes and compound hazards in a warming world. Annu. Rev. Earth Planet. Sci..

[2] Agarwal A (2019). Network-based identification and characterization of teleconnections on different scales. Sci. Rep..

[3] Seneviratne SI (2012). Changes in climate extremes and their impacts on the natural physical environment. Manag. Risks Extrem. Events Disasters..

[4] Shukla R, Chakraborty A, Sachdeva K, Joshi PK (2018). Agriculture in the western Himalayas: An asset turning into a liability. Dev. Pract..

[5] Shukla R (2021). Dynamic vulnerability of smallholder agricultural systems in the face of climate change for Ethiopia. Environ. Res. Lett..

[6] Sharma T, Vittal H, Karmakar S, Ghosh S (2020). Increasing agricultural risk to hydro-climatic extremes in India. Environ. Res. Lett..

[7] Kalyan A (2021). Multiscale spatiotemporal analysis of extreme events in the Gomati River Basin, India. Atmosphere.

[8] Mishra V, Thirumalai K, Singh D, Aadhar S (2020). Future exacerbation of hot and dry summer monsoon extremes in India. NPJ Clim. Atmos. Sci..

[9] Wehner M, Stone D, Krishnan H, AchutaRao K, Castillo F (2016). The deadly combination of heat and humidity in India and Pakistan in Summer 2015. Bull. Am. Meteorol. Soc..

[10] Bevacqua E, Maraun D, Hobæk Haff I, Widmann M, Vrac M (2017). Multivariate statistical modelling of compound events via pair-copula constructions: Analysis of floods in Ravenna (Italy). Hydrol. Earth Syst. Sci..

[11] Zscheischler J (2020). A typology of compound weather and climate events. Nat. Rev. Earth Environ..

[12] Leonard M (2014). A compound event framework for understanding extreme impacts. Wiley Interdiscip. Rev. Clim. Chang..

[13] Beniston M (2009). Trends in joint quantiles of temperature and precipitation in Europe since 1901 and projected for 2100. Geophys. Res. Lett..

[14] Trenberth KE, Shea DJ (2005). Relationships between precipitation and surface temperature. Geophys. Res. Lett..

[15] Hao Z, Singh VP, Hao F (2018). Compound extremes in hydroclimatology: A review. Water.

[16] Zscheischler J, Seneviratne SI (2017). Dependence of drivers affects risks associated with compound events. Sci. Adv..

[17] Sedlmeier K, Mieruch S, Schädler G, Kottmeier C (2016). Compound extremes in a changing climate: A Markov chain approach. Nonlinear Process. Geophys..

[18] Mueller B, Seneviratne SI (2012). Hot days induced by precipitation deficits at the global scale. Proc. Natl. Acad. Sci..

[19] Hao Z (2018). A multivariate approach for statistical assessments of compound extremes. J. Hydrol..

[20] Wu X, Hao Z, Zhang X, Li C, Hao F (2020). Evaluation of severity changes of compound dry and hot events in China based on a multivariate multi-index approach. J. Hydrol..

[21] Hao Z, Aghakouchak A, Phillips TJ (2013). Changes in concurrent monthly precipitation and temperature extremes. Environ. Res. Lett..

[22] Wu X, Hao Z, Hao F, Zhang X (2019). Variations of compound precipitation and temperature extremes in China during 1961–2014. Sci. Total Environ..

[23] Zscheischler J, Fischer E (2020). The record-breaking compound hot and dry 2018 growing season in Germany. Weather Clim. Extrem..

[24] Feng S, Hao Z (2020). Quantifying likelihoods of extreme occurrences causing maize yield reduction at the global scale. Sci. Total Environ..

[25] Toreti A, Cronie O, Zampieri M (2019). Concurrent climate extremes in the key wheat producing regions of the world. Sci. Rep..

[26] Wallemacq P, Below R, McLean D (2018). UNISDR and CRED Report: Economic Losses, Poverty & Disasters (1998–2017).

[27] Sharma S, Mujumdar P (2017). Increasing frequency and spatial extent of concurrent meteorological droughts and heatwaves in India. Sci. Rep..

[28] Kurths J (2019). Unravelling the spatial diversity of Indian precipitation teleconnections via a non-linear multi-scale approach. Nonlinear Process. Geophys..

[29] Satyanarayana P, Srinivas VV (2008). Regional frequency analysis of precipitation using large-scale atmospheric variables. J. Geophys. Res..

[30] Mannan A, Chaudhary S, Dhanya CT, Swamy AK (2018). Regionalization of rainfall characteristics in India incorporating climatic variables and using self-organizing maps. ISH J. Hydraul. Eng..

[31] Guntu RK, Maheswaran R, Agarwal A, Singh VP (2020). Accounting for temporal variability for improved precipitation regionalization based on self-organizing map coupled with information theory. J. Hydrol..

[32] Srivastava, A. K., Rajeevan, M. & Kshirsagar, S. R. Development of a high resolution daily gridded temperature data set (1969–2005) for the Indian region. *Atmos. Sci. Lett.* **10**, n/a-n/a (2009).

[33] Rajeevan M, Bhate J, Jaswal AK (2008). Analysis of variability and trends of extreme rainfall events over India using 104 years of gridded daily rainfall data. Geophys. Res. Lett..

[34] Subash N, Sikka AK (2014). Trend analysis of rainfall and temperature and its relationship over India. Theor. Appl. Climatol..

[35] Mondal A, Khare D, Kundu S (2015). Spatial and temporal analysis of rainfall and temperature trend of India. Theor. Appl. Climatol..

[36] IMD Website. http://www.imdpune.gov.in/Clim_Pred_LRF_New/Grided_Data_Download.html.

[37] Zhang X (2011). Indices for monitoring changes in extremes based on daily temperature and precipitation data: Indices for monitoring changes in extremes. Wiley Interdiscip. Rev. Clim. Chang..

[38] Sivakumar B, Woldemeskel FM (2015). A network-based analysis of spatial rainfall connections. Environ. Model. Softw..

[39] Sabeerali CT, Rao SA, Ajayamohan RS, Murtugudde R (2012). On the relationship between Indian summer monsoon withdrawal and Indo-Pacific SST anomalies before and after 1976/1977 climate shift. Clim. Dyn..

[40] Sahana AS, Ghosh S, Ganguly A, Murtugudde R (2015). Shift in Indian summer monsoon onset during 1976/1977. Environ. Res. Lett..

[41] Mann HB (1945). Nonparametric tests against trend. Econ. J. Econ. Soc..

[42] Kendall MG (1975). Rank correlation measures. Charles Griffin Lond..

[43] Taxak AK, Murumkar AR, Arya DS (2014). Long term spatial and temporal rainfall trends and homogeneity analysis in Wainganga basin, Central India. Weather Clim. Extrem..

[44] Guhathakurta P, Rajeevan M, Sikka DR, Tyagi A (2015). Observed changes in southwest monsoon rainfall over India during 1901–2011. Int. J. Climatol..

[45] Roxy MK (2017). A threefold rise in widespread extreme rain events over central India. Nat. Commun..

[46] Bollasina MA, Ming Y, Ramaswamy V (2011). Anthropogenic aerosols and the weakening of the south Asian summer monsoon. Science.

[47] Sandeep S, Ajayamohan RS, Boos WR, Sabin TP, Praveen V (2018). Decline and poleward shift in Indian summer monsoon synoptic activity in a warming climate. Proc. Natl. Acad. Sci. USA..

[48] Naidu CV (2015). An observational evidence of decrease in Indian summer monsoon rainfall in the recent three decades of global warming era. Glob. Planet. Change.

[49] Deshpande NR, Kothawale DR, Kulkarni A (2016). Changes in climate extremes over major river basins of India. Int. J. Climatol..

[50] Kumar V, Jain SK (2011). Trends in rainfall amount and number of rainy days in river basins of India (1951–2004). Hydrol. Res..

[51] Naidu CV (2009). Is summer monsoon rainfall decreasing over India in the global warming era?. J. Geophys. Res..

[52] Duhan D, Pandey A (2013). Statistical analysis of long term spatial and temporal trends of precipitation during 1901–2002 at Madhya Pradesh, India. Atmos. Res..

[53] Bisht DS, Chatterjee C, Raghuwanshi NS, Sridhar V (2018). Spatio-temporal trends of rainfall across Indian river basins. Theor. Appl. Climatol..

[54] Shekhar MS, Chand H, Kumar S, Srinivasan K, Ganju A (2010). Climate-change studies in the western Himalaya. Theor. Appl. Climatol..

[55] Shivam Goyal MK, Sarma AK (2017). Analysis of the change in temperature trends in Subansiri River basin for RCP scenarios using CMIP5 datasets. Theor. Appl. Climatol..

[56] Praveen B, Sharma P (2020). Climate change and its impacts on Indian agriculture: An econometric analysis. J. Public Aff..

[57] Kumar V, Jain SK, Singh Y (2010). Analysis of long-term rainfall trends in India. Hydrol. Sci. J..

[58] Jain SK, Kumar V (2012). Trend analysis of rainfall and temperature data for {India}. Curr. Sci..

[59] Kothawale DR, Kumar KR (2002). Tropospheric temperature variation over India and links with the Indian summer monsoon: 1971–2000. Mausam.

[60] Kothawale DR, Munot AA, Kumar KK (2010). Surface air temperature variability over India during 1901–2007, and its association with enso. Clim. Res..

[61] Guntu RK, Rathinasamy M, Agarwal A, Sivakumar B (2020). Spatiotemporal variability of Indian rainfall using multiscale entropy. J. Hydrol..

