# Supplementary material for: Disentangling increasing compound extremes at regional scale during Indian summer monsoon
Source: Sci Rep. 2021 Aug 12;11:16447. doi: 10.1038/s41598-021-95775-0 (PMC8360945; doi:10.1038/s41598-021-95775-0)
Supplement: Supplementary file 1 — Supplementary Information. [file 41598_2021_95775_MOESM1_ESM.docx]

**Supplementary Material**

**Disentangling increasing compound extremes at regional scale during Indian Summer Monsoon**

Ravi Kumar Guntu^1^ and Ankit Agarwal^1*^

^1^Department of Hydrology, Indian Institute of Technology Roorkee, 247667, India

*correspondence to: Ankit Agarwal ([ankit.agarwal@hy.iitr.ac.in](mailto:ankit.agarwal@hy.iitr.ac.in))

This document contains Appendix A, table and additional figures as mentioned in the main article. The figures provide details on the additional analysis carried out to reach the conclusions. List of table and figures in order of their presentation are as follows:

[**Table S1: Number of grid points for each homogenous region** 5](#_Toc72243799)

[**Figure S1: Frequency of CDHE’s (a-d) and CWCE’s (e-f) for ten homogenous regions during Indian summer months for the time duration 1951-1976.** **This figure is generated using Mapping Toolbox in MATLAB R2020b (Version 9.9 https://in.mathworks.com/products/mapping.html)** 6](#_Toc72243844)

[**Figure S2: Frequency of CDHE’s (a-d) and CWCE’s (e-f) for ten homogenous regions during Indian summer months for the time duration 1977-2019.** **This figure is generated using Mapping Toolbox in MATLAB R2020b (Version 9.9 https://in.mathworks.com/products/mapping.html)** 6](#_Toc72243845)

[**Figure S3: Temporal variation of dry/hot spatial extent for a) Western India b) North-western India c) North-central India d) Eastern India e) South-central India f) South eastern coastline g) Konkan coast h) North-eastern India i) Rain-belt Western Himalayan and j) Rain-shadow Western Himalayan during June, July, August and September for the study period 1951-2019.** **This figure is generated in MATLAB R 2020b (Version 9.9 https://in.mathworks.com)** 9](#_Toc72243846)

[**Figure S4: Temporal variation of wet/cold spatial extent for a) Western India b) North-western India c) North-central India d) Eastern India e) South-central India f) South eastern coastline g) Konkan coast h) North-eastern India i) Rain-belt Western Himalayan and j) Rain-shadow Western Himalayan during June, July, August and September for the study period 1951-2019.** **This figure is generated in MATLAB R 2020b (Version 9.9 https://in.mathworks.com)** 11](#_Toc72243847)

[**Figure S5: Geographical representation of spatial extent for Western India during a) June, b) July, c) August and d) September at decadal time scale. Colorbar represents the frequency of dry/hot per decade. This figure is generated using Mapping Toolbox in MATLAB R2020b (Version 9.9 https://in.mathworks.com/products/mapping.html)** 12](#_Toc72243848)

[**Figure S6: Same as Figure S5 for North-western India. This figure is generated using Mapping Toolbox in MATLAB R2020b (Version 9.9 https://in.mathworks.com/products/mapping.html)** 12](#_Toc72243849)

[**Figure S7: Same as Figure S5 for Eastern India.** **This figure is generated using Mapping Toolbox in MATLAB R2020b (Version 9.9 https://in.mathworks.com/products/mapping.html)** 13](#_Toc72243850)

[**Figure S8: Same as Figure S5 for South-central India.** **This figure is generated using Mapping Toolbox in MATLAB R2020b (Version 9.9 https://in.mathworks.com/products/mapping.html)** 13](#_Toc72243851)

[**Figure S9: Same as Figure S5 for South-eastern coastline.** **This figure is generated using Mapping Toolbox in MATLAB R2020b (Version 9.9 https://in.mathworks.com/products/mapping.html)** 14](#_Toc72243852)

[**Figure S10: Same as Figure S5 for Konkan coast.** **This figure is generated using Mapping Toolbox in MATLAB R2020b (Version 9.9 https://in.mathworks.com/products/mapping.html)** 15](#_Toc72243853)

[**Figure S11: Same as Figure S5 for North-eastern India.** **This figure is generated using Mapping Toolbox in MATLAB R2020b (Version 9.9 https://in.mathworks.com/products/mapping.html)** 16](#_Toc72243854)

[**Figure S12: Geographical representation of spatial extent for Western India during a) June, b) July, c) August and d) September at decadal time scale. Colorbar represents the frequency of wet/cold per decade.** **This figure is generated using Mapping Toolbox in MATLAB R2020b (Version 9.9 https://in.mathworks.com/products/mapping.html)** 16](#_Toc72243855)

[**Figure S13: Same as Figure S12 for North-western India.** **This figure is generated using Mapping Toolbox in MATLAB R2020b (Version 9.9 https://in.mathworks.com/products/mapping.html)** 17](#_Toc72243856)

[**Figure S14: Same as Figure S12 for North-central India.** **This figure is generated using Mapping Toolbox in MATLAB R2020b (Version 9.9 https://in.mathworks.com/products/mapping.html)** 17](#_Toc72243857)

[**Figure S15: Same as Figure S12 for Eastern India.** **This figure is generated using Mapping Toolbox in MATLAB R2020b (Version 9.9 https://in.mathworks.com/products/mapping.html)** 18](#_Toc72243858)

[**Figure S16: Same as Figure S12 for South-central India.** **This figure is generated using Mapping Toolbox in MATLAB R2020b (Version 9.9 https://in.mathworks.com/products/mapping.html)** 18](#_Toc72243859)

[**Figure S17: Same as Figure S12 for South-eastern coastline.** **This figure is generated using Mapping Toolbox in MATLAB R2020b (Version 9.9 https://in.mathworks.com/products/mapping.html)** 19](#_Toc72243860)

[**Figure S18: Same as Figure S12 for Konkan coast.** **This figure is generated using Mapping Toolbox in MATLAB R2020b (Version 9.9 https://in.mathworks.com/products/mapping.html)** 20](#_Toc72243861)

[**Figure S19: Empirical CDF of dry/hot (a-d) and wet/cold (e-f) spatial extent during June, July, August and September for the time windows 1951-1976 and 1977-2019 for Western India.** **This figure is generated in MATLAB R 2020b (Version 9.9 https://in.mathworks.com)** 21](#_Toc72243862)

[**Figure S20: Same as Figure S19 for North-western India.** **This figure is generated in MATLAB R 2020b (Version 9.9 https://in.mathworks.com)** 21](#_Toc72243863)

[**Figure S21: Same as Figure S19 for Eastern India.** **This figure is generated in MATLAB R 2020b (Version 9.9 https://in.mathworks.com)** 22](#_Toc72243864)

[**Figure S22: Same as Figure S19 for South-central India.** **This figure is generated in MATLAB R 2020b (Version 9.9 https://in.mathworks.com)** 22](#_Toc72243865)

[**Figure S23: Same as Figure S19 for South-eastern coastline.** **This figure is generated in MATLAB R 2020b (Version 9.9 https://in.mathworks.com)** 23](#_Toc72243866)

[**Figure S24: Same as Figure S19 for Konkan coast.** **This figure is generated in MATLAB R 2020b (Version 9.9 https://in.mathworks.com)** 23](#_Toc72243867)

[**Figure S25: Same as Figure S19 for north-eastern India.** **This figure is generated in MATLAB R 2020b (Version 9.9 https://in.mathworks.com)** 24](#_Toc72243868)

[**Figure S26: Same as Figure S19 for Rain belt Western Himalayan.** **This figure is generated in MATLAB R 2020b (Version 9.9 https://in.mathworks.com)** 24](#_Toc72243869)

[**Figure S27:Same as Figure S19 for Rain shadow Western Himalayan.** **This figure is generated in MATLAB R 2020b (Version 9.9 https://in.mathworks.com)** 25](#_Toc72243870)

**TEXT**

**S.1: Mann-Kendall test**

The MK test is a non-parametric test that considers the test statistic, S to have zero mean and variance estimated as

$S=\sum_{i=1}^{n-1} \sum_{j=i+1}^{n} \mathrm{sgn}(x_{j}-x_{i})$ (1)

$\mathrm{sgn}(x_{j}-x_{i})=\left\{\begin{matrix} +1, if x_{j}-x_{i}>0 \\ 0, if x_{j}-x_{i}=0 \\ -1 if x_{j}-x_{i}<0 \end{matrix}\right.$ (2)

$Var(S)=\frac{n(n-1)(2n+5)-\sum_{t=1}^{m} t_{i}(t_{i}-1)(2t_{i}+5)}{18}$ (3)

where n is the number of data points, m is the number of tied groups; t denotes the number of ties of extent *i*, $x_{i}$ and $x_{j}$ are the data values in the time series at *i*^th^ and *j*^th^ data point (such that *j* > *i*), respectively, and $\mathrm{sgn}(x_{j}-x_{i})$ is the sign function. In cases where the sample size *n* > 10, the standard normal variable *Z* is computed as,

$Z=\left\{\begin{matrix} \frac{S-1}{\sqrt{Var(S)}} if S>0 \\ 0 if S=0 \\ \frac{S+1}{\sqrt{Var(S)}} if S<0 \end{matrix}\right.$ (4)

Positive values of *Z* indicate increasing trends, while negative values imply decreasing trends in the time series. The null hypothesis of the test says there is no trend in the time series of spatial extent. The null hypothesis of no trend is rejected for an absolute value of *Z* greater than $Z_{1-\alpha/2}$, obtained from the standard normal cumulative distribution tables. In this study, a significance level of $\alpha=0.05$ is used.

**S.2: Two-sample Kolmogorov-Smirnov Test**

The two-sample Kolmogorov-Smirnov (KS) tests whether two samples come from the same or different distribution family. KS is a non-parametric test that can evaluate two distribution functions (for two different samples) based on the distance between their empirical distribution functions. The null hypothesis is that the two distribution functions are drawn from the same distribution at a given significance level (here, α = 0.05). Here, the two-sample KS test is employed to assess the differences between the spatial distribution of the CDHE and CWCE characteristics evaluated for the recent period (1977-2019) and base period (1951-1976) ten homogenous regions. The test indicates whether the data from the two periods come from the same distribution at a 95% confidence level.

For two given samples, one with a sample size of m and a cumulative distribution function (CDF) of F(x) and the other with a sample size of n and a CDF of G(x). The maximum distance between the two CDFs can be given as,

$$D_{m,n}=\max_{x} |F(x)-G(x)|$$

As for the Kolmogorov-Smirnov test for normality, the null hypothesis of same distribution (at significance level α) is rejected if D_m,n_ > D_m,n,α_ where D_m,n,α_ is the critical value with m and n being sufficiently large and *D_m_*,*_n_*, _α_ given by

$$D_{m,n,\alpha}=c(\alpha)\sqrt{\frac{m+n}{mn}}$$

where c(α) = the inverse of the Kolmogorov distribution at α

**Table S1: Number of grid points for each homogenous region**

| **No** | **Homogenous region** | **Total number of Grid points** |
| --- | --- | --- |
| 1 | Western India | 18 |
| 2 | North-western India | 50 |
| 3 | North-central India | 65 |
| 4 | Eastern India | 36 |
| 5 | South-central India | 44 |
| 6 | South-eastern coastline | 14 |
| 7 | Konkan Coast | 9 |
| 8 | North-eastern India | 21 |
| 9 | Rain-belt Western Himalayan | 17 |
| 10 | Rain-shadow Western Himalayan | 10 |

**Figure S1: Frequency of CDHE’s (a-d) and CWCE’s (e-f) for ten homogenous regions during Indian summer months for the time duration 1951-1976.** **This figure is generated using Mapping Toolbox in MATLAB R2020b (Version 9.9 https://in.mathworks.com/products/mapping.html)**

**Figure S2: Frequency of CDHE’s (a-d) and CWCE’s (e-f) for ten homogenous regions during Indian summer months for the time duration 1977-2019.** **This figure is generated using Mapping Toolbox in MATLAB R2020b (Version 9.9** [**https://in.mathworks.com/products/mapping.html**](https://in.mathworks.com/products/mapping.html)**)**

| **a)** | **b)** |
| --- | --- |
| **c)** | **d)** |
| **e)** | **f)** |
| **g)** | **h)** |
| **i)** | **j)** |

**Figure S3: Temporal variation of dry/hot spatial extent for a) Western India b) North-western India c) North-central India d) Eastern India e) South-central India f) South eastern coastline g) Konkan coast h) North-eastern India i) Rain-belt Western Himalayan and j) Rain-shadow Western Himalayan during June, July, August and September for the study period 1951-2019.** **This figure is generated in MATLAB R 2020b (Version 9.9 https://in.mathworks.com)**

| **a)** | **b)** |
| --- | --- |
| **c)** | **d)** |
| **e)** | **f)** |
| **g)** | **h)** |
| **i)** | **j)** |

**Figure S4: Temporal variation of wet/cold spatial extent for a) Western India b) North-western India c) North-central India d) Eastern India e) South-central India f) South eastern coastline g) Konkan coast h) North-eastern India i) Rain-belt Western Himalayan and j) Rain-shadow Western Himalayan during June, July, August and September for the study period 1951-2019.** **This figure is generated in MATLAB R 2020b (Version 9.9 https://in.mathworks.com)**

**Figure S5: Geographical representation of spatial extent for Western India during a) June, b) July, c) August and d) September at decadal time scale. Colorbar represents the frequency of dry/hot per decade. This figure is generated using Mapping Toolbox in MATLAB R2020b (Version 9.9 https://in.mathworks.com/products/mapping.html)**

**Figure S6: Same as Figure S5 for North-western India. This figure is generated using Mapping Toolbox in MATLAB R2020b (Version 9.9 https://in.mathworks.com/products/mapping.html)**

**Figure S7: Same as Figure S5 for Eastern India.** **This figure is generated using Mapping Toolbox in MATLAB R2020b (Version 9.9 https://in.mathworks.com/products/mapping.html)**

**Figure S8: Same as Figure S5 for South-central India.** **This figure is generated using Mapping Toolbox in MATLAB R2020b (Version 9.9 https://in.mathworks.com/products/mapping.html)**

**Figure S9: Same as Figure S5 for South-eastern coastline.** **This figure is generated using Mapping Toolbox in MATLAB R2020b (Version 9.9 https://in.mathworks.com/products/mapping.html)**

**Figure S10: Same as Figure S5 for Konkan coast.** **This figure is generated using Mapping Toolbox in MATLAB R2020b (Version 9.9 https://in.mathworks.com/products/mapping.html)**

**Figure S11: Same as Figure S5 for North-eastern India.** **This figure is generated using Mapping Toolbox in MATLAB R2020b (Version 9.9 https://in.mathworks.com/products/mapping.html)**

**Figure S12: Geographical representation of spatial extent for Western India during a) June, b) July, c) August and d) September at decadal time scale. Colorbar represents the frequency of wet/cold per decade.** **This figure is generated using Mapping Toolbox in MATLAB R2020b (Version 9.9 https://in.mathworks.com/products/mapping.html)**

**Figure S13: Same as Figure S12 for North-western India.** **This figure is generated using Mapping Toolbox in MATLAB R2020b (Version 9.9 https://in.mathworks.com/products/mapping.html)**

**Figure S14: Same as Figure S12 for North-central India.** **This figure is generated using Mapping Toolbox in MATLAB R2020b (Version 9.9 https://in.mathworks.com/products/mapping.html)**

**Figure S15: Same as Figure S12 for Eastern India.** **This figure is generated using Mapping Toolbox in MATLAB R2020b (Version 9.9 https://in.mathworks.com/products/mapping.html)**

**Figure S16: Same as Figure S12 for South-central India.** **This figure is generated using Mapping Toolbox in MATLAB R2020b (Version 9.9 https://in.mathworks.com/products/mapping.html)**

**Figure S17: Same as Figure S12 for South-eastern coastline.** **This figure is generated using Mapping Toolbox in MATLAB R2020b (Version 9.9 https://in.mathworks.com/products/mapping.html)**

**Figure S18: Same as Figure S12 for Konkan coast.** **This figure is generated using Mapping Toolbox in MATLAB R2020b (Version 9.9 https://in.mathworks.com/products/mapping.html)**

**Figure S19: Empirical CDF of dry/hot (a-d) and wet/cold (e-f) spatial extent during June, July, August and September for the time windows 1951-1976 and 1977-2019 for Western India.** **This figure is generated in MATLAB R 2020b (Version 9.9 https://in.mathworks.com)**

**Figure S20: Same as Figure S19 for North-western India.** **This figure is generated in MATLAB R 2020b (Version 9.9 https://in.mathworks.com)**

**Figure S21: Same as Figure S19 for Eastern India.** **This figure is generated in MATLAB R 2020b (Version 9.9 https://in.mathworks.com)**

**Figure S22: Same as Figure S19 for South-central India.** **This figure is generated in MATLAB R 2020b (Version 9.9 https://in.mathworks.com)**

**Figure S23: Same as Figure S19 for South-eastern coastline.** **This figure is generated in MATLAB R 2020b (Version 9.9 https://in.mathworks.com)**

**Figure S24: Same as Figure S19 for Konkan coast.** **This figure is generated in MATLAB R 2020b (Version 9.9 https://in.mathworks.com)**

**Figure S25: Same as Figure S19 for north-eastern India.** **This figure is generated in MATLAB R 2020b (Version 9.9 https://in.mathworks.com)**

**Figure S26: Same as Figure S19 for Rain belt Western Himalayan.** **This figure is generated in MATLAB R 2020b (Version 9.9 https://in.mathworks.com)**

**Figure S27:Same as Figure S19 for Rain shadow Western Himalayan.** **This figure is generated in MATLAB R 2020b (Version 9.9 https://in.mathworks.com)**
